# Early Adverse Events, HPA Activity and Rostral Anterior Cingulate Volume in MDD

**DOI:** 10.1371/journal.pone.0004887

**Published:** 2009-03-26

**Authors:** Michael T. Treadway, Merida M. Grant, Zhaohua Ding, Steven D. Hollon, John C. Gore, Richard C. Shelton

**Affiliations:** 1 Department of Psychology, Vanderbilt University, Nashville, Tennessee, United States of America; 2 Vanderbilt University Institute of Imaging Sciences, Department of Radiology and Radiological Sciences, Vanderbilt University Medical Center, Nashville, Tennessee, United States of America; 3 Department of Psychiatry, Vanderbilt University Medical Center, Nashville, Tennessee, United States of America; James Cook University, Australia

## Abstract

**Background:**

Prior studies have independently reported associations between major depressive disorder (MDD), elevated cortisol concentrations, early adverse events and region-specific decreases in grey matter volume, but the relationships among these variables are unclear. In the present study, we sought to evaluate the relationships between grey matter volume, early adverse events and cortisol levels in MDD.

**Methods/Results:**

Grey matter volume was compared between 19 controls and 19 individuals with MDD using voxel-based morphometry. A history of early adverse events was assessed using the Childhood Trauma Questionnaire. Subjects also provided salivary cortisol samples. Depressed patients showed decreased grey matter volume in the rostral ACC as compared to controls. Rostral ACC volume was inversely correlated with both cortisol and early adverse events.

**Conclusions:**

These findings suggest a key relationship between ACC morphology, a history of early adverse events and circulating cortisol in the pathophysiology of MDD.

## Introduction

Early adverse events are a significant risk factor for the subsequent development of major depressive disorder (MDD) [1]. It has been theorized that one neurobiological mechanism through which early adverse events may elevate the risk of developing depression is by increased activation of the hypothalamic-pituitary-adrenal (HPA) axis, a key stress-hormone signaling pathway. This hypothesis is derived from two areas of research. First, both animal and clinical models suggest that depression is associated with poor regulation of HPA axis activity, as indicated by elevated cortisol [2], disruption of circadian HPA rhythms [2], [3], and failure to suppress cortisol levels following administration of the synthetic steroid dexamethasone [3]. Second, preclinical studies have revealed that prolonged exposure to glucocorticoids is associated with atrophy of brain regions involved in the regulation of HPA activity, including the hippocampus [4] and the medial prefrontal cortex (mPFC) [5]. Stress-related damage to these regulatory regions may precipitate a cycle of diminished resiliency, in which the system is less able to regulate HPA activity in response to future stress, resulting in greater exposure to glucocorticoids.

Considerable prior research has focused on the hippocampus, given its well-established role in regulating HPA activity, and the high density of glucocorticoid receptors expressed in this region [4]. However, more recent work has also emphasized a prominent role for the mPFC, particularly the anterior cingulate cortex (ACC). The ACC contains high concentrations of glucocorticoid receptors (GR) in laminas II, III, and V [6], is vulnerable to the noxious effects of glucocorticoids [7], [8], and may exert inhibitory control over the HPA axis via GR-mediated negative-feedback [6], [9]. Consistent with these preclinical findings, human neuroimaging studies of individuals with MDD have reported volumetric reductions in the hippocampus [10]–[15] and the ACC [14], [16], [17].

Evidence for stress-related structural damage to the anterior cingulate is particularly relevant for understanding the relationship between stress and depression, as significant research suggests that dysregulation within cortico-limbic pathways involving the ACC may be responsible for impairments in cognition, emotion and motivation in the disorder [18], [19]. Importantly, hypoactivity in the dorsal subdivision (dACC) [20], and hyperactivity in the rostral subdivision (rACC) [21] have been demonstrated in MDD, along with impaired ACC-amygdala connectivity [22].

## Methods

### Objectives

In the present study, we sought to examine the relationship between early adverse events, HPA activity and grey matter volume among individuals with unipolar depression and healthy controls, with a particular emphasis on grey matter volume of the hippocampus and anterior cingulate. By assessing all three variables within a single sample, the present study is able to provide a more direct evaluation of the putative neurobiological mechanisms that are believed to underlie the observed volumetric decreases in HPA-axis regulatory regions in MDD.

### Participants

Nineteen patients with current depression and nineteen healthy matched control subjects were recruited for this study. All participants were between 18 and 55 years of age with no significant history of neurological disease or lifetime history of brain injury, psychosis, mania, or substance dependence. Additionally, participants were excluded if they reported substance abuse within the previous six months. All patients were diagnosed with unipolar depression and met full criteria for one or more episodes of major depressive disorder as determined by a Structured Clinical Interview (SCID) for DSM-IV. Patients were excluded if they met criteria for specific comorbid Axis I disorders that included alcohol dependence, obsessive-compulsive disorder, schizophrenia and other psychotic disorders or bipolar disorder. In addition, all patients had a score of 16 or higher on the Hamilton Depression Rating Scale (HDRS) [23]. The presence of Axis-II psychopathology was not assessed. Patients were antidepressant-free at the time of scanning. Never-depressed control subjects did not meet criteria for any Axis I mood disorders except for one subject who was diagnosed with mild agoraphobia without panic disorder as determined by the SCID. All never-depressed control subjects had a score of six or less on the HDRS. Subjects who met criteria were scheduled for a scan session within one week of admission to the study.

### Ethics Statement

The Vanderbilt University Institutional Review Board approved the experimental protocol. A complete description of the study was provided to all participants, and all subjects provided written informed consent. Subjects were recruited through the Vanderbilt University Medical Center Outpatient Psychiatry Clinic or through television advertisements.

### Behavioral Measures

To assess a history of early adverse events, participants completed the Childhood Trauma Questionnaire – Short Form (CTQ-SF) [24]. The CTQ-SF was developed as a 28-item questionnaire derived from the original 70-item Childhood Trauma Questionnaire. The CTQ-SF has 25 clinical questions and three validity items. The measure has five sub-scales comprised of five questions each that assess childhood maltreatment in the areas of emotional, physical, or sexual abuse, emotional neglect and physical neglect. Subjects rate statements about childhood lifetime experiences on a five-point scale (“never true” to “very often true”). Items are generally stated in objective terms, (e.g., “When I was growing up, someone touched me in a sexual way or made me touch them”), whereas some items require subjective evaluation (e.g., “When I was growing up, I believe I was sexually abused”) and usually do not specify the relationship of the perpetrator(s) to the subject. Emotional abuse items are general (e.g., “People in my family said hurtful or insulting things to me”) but do not investigate the specific verbal content of the abuse. Reliability and validity of the CTQ, including its stability over time, convergent and discriminant validity with structured trauma interviews, and corroboration using independent data, have been determined. The CTQ-SF has demonstrated high internal reliability, with (Cronbach's alphas ranging from .74 to .90) and good test-retest reliability at three months (r = .80). Scores on each subscale were calculated by taking the mean value of the five individual items for each subscale. Scores of patients and controls subscales were compared using an independent samples t-test, assuming unequal variance. A Bonferroni correction was applied to control for multiple comparisons (corrected α = .01).

### Salivary Cortisol

Samples of saliva were collected using the Salivette saliva collection device (Sarstedt, Newton NC). Participants collected three saliva samples per day for two consecutive days and a sample on the morning of their MRI scan session on the third day. The first sample was recorded within 0.5 h after awakening. Two additional samples were collected at 3:00 PM and 9:00 PM. Using this method we ensured that we could adequately account for diurnal variation. Cortisol levels were determined using an enzyme immunoassay (ALPCO Diagnostics, Salem, NH). For all subsequent analyses, the average of all seven cortisol samples was used unless otherwise specified.

### Structural MRI Image Acquisition

Structural MRI scans were acquired on a 3T Philips Intera Achieva scanner at the Vanderbilt University Institute of Imaging Sciences (VUIIS). High-resolution structural images were acquired in the axial plane to facilitate spatial normalization using a 3D IR Prepped 3DFFE sequence (TR = 10.1 ms, TE = 4.2 ms, FOV = 24×24 cm2, matrix size = 256×256, slice thickness = l.2 mm, no gap). Due to scanner error, 3D data were not available for one subject. For this subject we used a 2D imaging sequence (TR = 450 ms, TE = 17 ms, FOV = 24×24 cm2, matrix size = 256×256, slice thickness = 4 mm, no gap). Data for this subject were re-interpolated into a 3D image matrix (matrix size 256×256). Exclusion of this subject did not alter the results.

### Voxel-Based Morphometry

Data were analyzed on a Dell Vostro 200 (Dell Inc, Round Rock, Tx) running a Linux-based operating system (Ubuntu 7.1). Voxel-based morphometry (VBM) was performed using MATLAB7.4.0 (Mathworks, Natick, MA) and SPM2 (Wellcome Department of Imaging Neuroscience, London, UK). All VBM analyses strictly adhered to the optimized VBM protocol as described by Good et al (2001) [25]. All structural images were examined for artifacts and then reoriented to a center point located on the anterior commissure. A customized anatomical template was created from the reoriented structural MRI images of all subjects. Template creation included spatial normalization of all the images to MNI space. The customized template was then used in conjunction with SPM prior images for grey matter, white matter and cerebrospinal fluid (CSF) as the basis for spatial normalization for all subjects. Spatially normalized images were then re-sliced with a final voxel size of 1.5×1.5×1.5 mm^3^, and were subsequently segmented by compartment into grey matter, white matter, and CSF images. After segmentation, the segmented grey matter images were modulated by multiplication of the Jacobian determinant of the spatial normalization function, so as to allow for the estimation of volumetric differences between groups [25]. Images were then smoothed using a 12-mm FWHM isotropic Gaussian kernel. All subsequent statistical analyses were performed on the normalized, segmented, modulated and smoothed grey matter images.

### Group level analysis

Group differences between patients and controls were assessed using an ANCOVA model as implemented in SPM2, with age, sex and total intracranial volume used as covariates. Total intracranial volume was calculated as the sum of segmented gray, white and CSF images for each subject.

Volumetric differences between groups were evaluated using both a whole-brain, and region of interest (ROI) approach. Whole-brain analyses were conducted using a family-wise error correction of p_FWE_<.05 to control for multiple comparisons. *A priori* ROI included the anterior cingulate (dorsal and rostral ACC [BA 24], [Bibr pone.0004887-Wagner1] [32 and 25]) and the hippocampus. These regions were selected because both have shown evidence of atrophy in MDD [10]–[17], and preclinical findings have suggested that both regions are involved in regulating HPA activity [4], [5], and are damaged by elevated exposure from glucocorticoids resulting from chronic stress [4], [7]–[9].

ROI for the anterior cingulate and hippocampus were derived from the Anatomical Automatic Labeling atlas [26] as implemented in the Wake Forest University Pickatlas [27]. Separate masks were drawn for the rostral and dorsal portions of the anterior cingulate, consistent with prior findings suggesting that these sub-regions are differently affected in MDD [19], [20]. All reported clusters were corrected for multiple comparisons using a family-wise error correction of p_FWE_<.05.

### Correlations between Volume, Cortisol and Early Life Stress

Once statistically significant clusters were identified, estimates of grey matter volume from each cluster were extracted from SPM and entered into SPSS (SPSS for Windows, Rel. 15.0. 2006. Chicago: SPSS Inc.) for further analysis. All SPSS analyses were conducted on a Dell Dimension workstation (Dell, Round Rock, TX), running Windows XP (Microsoft, Redmond, WA). Separate analyses were used to explore the relationship between GM volume (within regions identified from group SPM comparisons), the CTQ combined physical/sexual abuse scale, and total average cortisol for patients and for controls. Partial correlations were used to control for the effects of age and sex within each group.

## Results

### Sample Characteristics

Participants in the study included 19 patients diagnosed with major depression (female = 10) and 19 age, gender and IQ matched healthy volunteers (female = 10). A summary of subject characteristics is presented in Table 1.

**Table 1:** Demographic Data, CTQ Scores and Salivary Cortisol.

Variable	MDD			Healthy Controls		
	*n*	*Mean*	*SD*	*n*	*Mean*	*SD*
Number of female participants	10			10		
Age	19	35.2	10.5	19	30.3	8.6
Estimated IQ (Shipley)	19	105.6	7.8	19	108.4	9.9
Hamilton Rating Scale of Depression**	19	21.5	4.1	19	0.84	1.3
Number of previous episodes	19	2.6	0.9	0		
Average duration of illness (years)	19	12.9	13.7	0		
Past alcohol abuse	2			0		
Co-morbid anxiety disorder	7			0		
Past anxiety disorder	3			1		
†CTQ Emotional Abuse Scale**	19	11.7	6.1	14	5.6	0.7
CTQ Physical Abuse Scale**	19	10.2	4.5	14	5.4	0.7
CTQ Sexual Abuse Scale**	19	10.0	6.9	14	5.0	0
CTQ Emotional Neglect**	19	12.3	4.2	14	6.8	1.9
CTQ Physical Neglect**	19	8.6	4.0	14	5.1	0.36
CTQ Physical and Sexual Abuse Scale**	19	10.1	5.4	14	5.2	0.32
†Salivary Cortisol (morning samples)*	16	10.9	3.5	15	8.2	2.9
Salivary Cortisol (all samples)*	16	7.6	2.0	15	6.3	1.9

* *p<.05*.

** *p<.01*.

† outliers and missing data have been excluded.

### CTQ Results

CTQ data were not available for four of the nineteen control subjects, who were recruited prior to the inclusion of the CTQ in the study protocol. Additionally, one control subject was a statistical outlier (Z-score>3), and was excluded. All patients with MDD completed the questionnaire. Patients with unipolar depression had significantly higher scores on all five scales after correcting for multiple comparisons (Table 1).

### Salivary Cortisol Results

**Figure 1:**
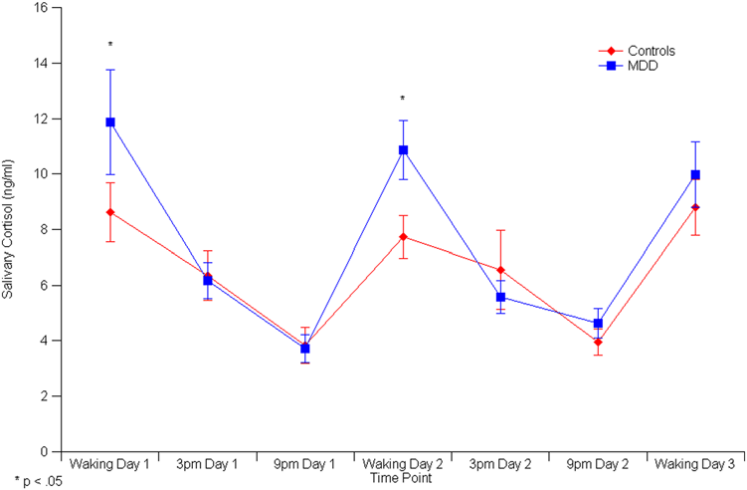
Time course of salivary cortisol (ng/ml) for patients and controls.

Due to insufficient saliva concentrations, accurate cortisol estimates were unavailable for three control subjects and three patients. Additionally, one of the control subjects was an outlier and was excluded from subsequent analysis. Using a one-sample t-test, it was found that average cortisol levels (the sum of all samples divided by seven) for the patient group were elevated when compared to the control group (t_29_ = −1.76, p = .045). However, differences in cortisol secretion between the patient and control groups were greatest for average morning cortisol (t_29_ = −2.32, p = .014) (Table 1) (Figure 1).

### Voxel-Based Morphometry Results – Whole Brain Analysis

There were no significant differences between patients and controls using a whole brain analysis, correcting for multiple comparisons at an alpha set to p_FWE_<.05.

### Voxel-Based Morphometry Results – ROI Analysis

**Figure 2:**
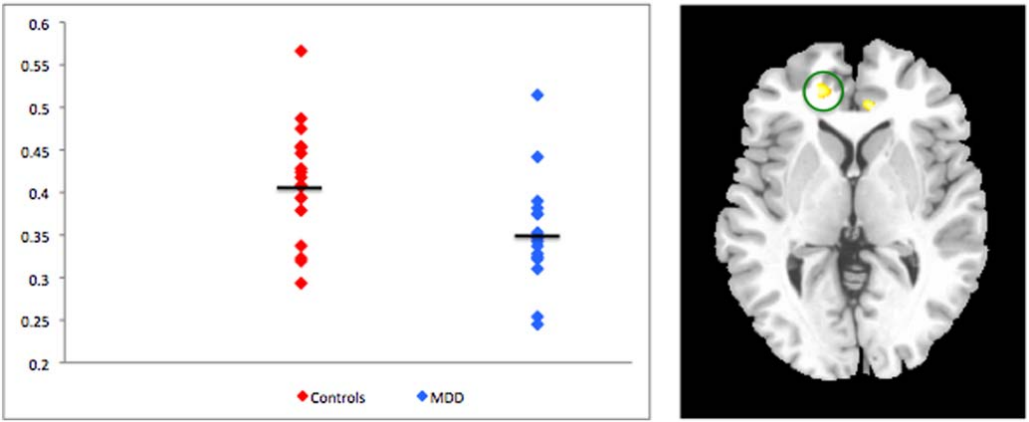
Differences in volume of rACC among controls and patients with MDD. Brain image is masked at an uncorrected threshold at p<.01.

ROI analysis of volumetric differences between patients and controls revealed an area of decreased volume in the MDD group in the right rostral ACC, BA 32; [x = 17 y = 44 z = 1] (t_33_ = 3.86, p_FWE_ = .041). A second cluster of decreased volume in the MDD group appeared in the left rostral ACC, BA 24 [x = −9 y = 38 z = 0]. This cluster was only marginally significant after correcting for multiple comparisons (t_33_ = 3.54, p_FWE_ = .083) (Figure 2). No differences between patients and controls were found for the hippocampus at uncorrected thresholds of either p = .001, or p = .01.

### Correlations between Volume, Cortisol and Early Life Stress

**Figure 3:**
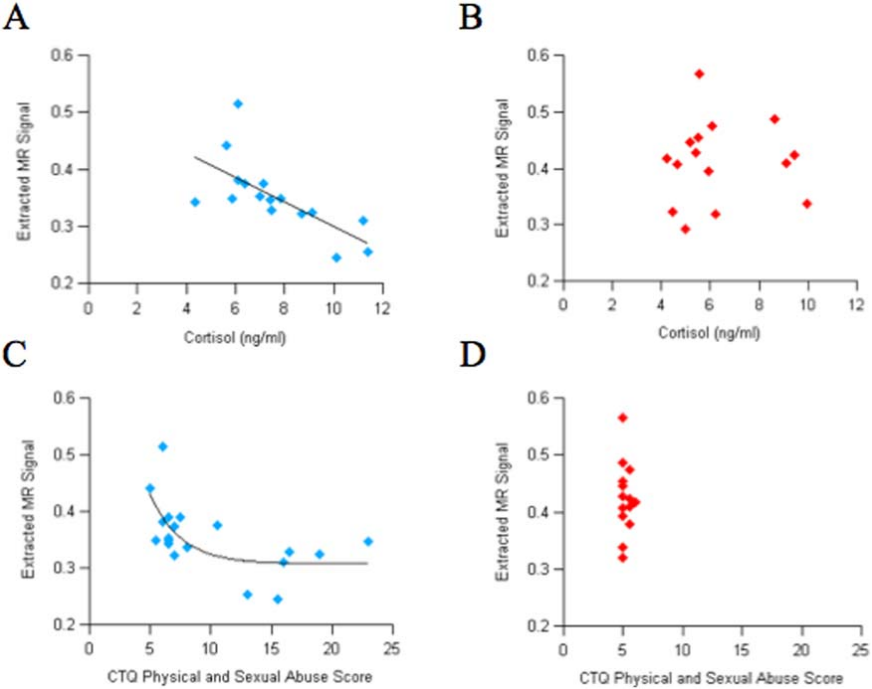
Correlations between volumetric decrease in rACC and average cortisol levels for patients (A) and controls (B) and correlations between volumetric decrease in rACC and the combined CTQ scales for physical and sexual abuse for patients (C) and controls (D).

Estimates of grey matter volume were extracted from all voxels that survived an uncorrected threshold of p = .01 of the right rACC cluster. Within the patient group, individual differences in rACC volume showed a significant inverse correlation using an inverse curve fit with the combined CTQ sexual and physical abuse scale (r = −.62, p = .004). This correlation remained significant after controlling for age (r = −.56, p = .015) and sex (r = −.68, p = .002). Average salivary cortisol levels were also inversely correlated with volume in the rACC (r = −.69, p = .003). This correlation remained significant after controlling for age (r = −.57, p = .028) and sex (r = −.72, p = .002). Among the control group, there were no significant correlations between rACC and the CTQ subscales or cortisol (Table 2) (Figure 3).

**Table 2:** Correlations between volume, cortisol and CTQ scores.

Correlations with right rACC cluster		*n*	r	p
MDD Group				
	CTQ physical and sexual abuse - combined†	19	−0.62	0.004
	Cortisol (all samples)†	16	−0.69	0.003
Control Group				
	CTQ physical and sexual abuse - combined	14	0.10	0.74
	Cortisol (all samples)	15	0.02	0.94

† correlations remained significant after controlling for age and sex.

## Discussion

The present study found that individuals with MDD demonstrated a volumetric decrease in the right rACC (BA 32) as compared to controls. This finding is supported in part by previous studies, which showed that grey matter volume within other sub-regions of the ACC is reduced in individuals with MDD [14], [16], [17]. Notably, our findings demonstrate linkages between decreased rACC volume in the disorder and both salivary cortisol and early childhood maltreatment. These data extend findings from preclinical studies suggesting that observed volumetric decreases in the ACC may be a consequence of prolonged exposure to glucocorticoids resulting from chronic stress [7], [8].

Localization of the decrease in anterior cingulate volume to the rostral subdivision is particularly relevant, as this region has been strongly implicated in the pathophysiology of depression in previous studies [19], [21], [28]–[33]. Additionally, several fMRI studies using working memory and attention tasks have reported that individuals with current or remitted MDD exhibit increased rACC activity in order to match the same level of performance as control subjects [31]–[33]. This suggests that the rACC may be less efficient in individuals with MDD as compared to controls, which may result from altered ACC morphology. Consistent with this interpretation is a recent animal study that found reduced working memory performance and decreased connectivity between the hippocampus and ACC following exposure to chronic stress, which was accompanied by atrophy in laminas I and II of the ACC [34].

Prior reports of decreased cingulate volume have emphasized changes in the left hemisphere (14, 17), although some reports have suggested decreased cingulate volume bilaterally [35], [16]. Additionally, a recent meta-analysis of volumetric changes in depression demonstrated that both right and left anterior cingulate volumes are reduced in the disorder [36]. In our study, although we emphasize the volumetric decrease in right anterior cingulate, which remained significant after correcting for multiple comparisons, a small cluster in the left hemisphere was also marginally significant.

The observed relationship between reduced rACC volume and a history of early adverse events is consistent with prior findings that have revealed a relationship between chronic and repeated stress and anterior cingulate structure [7], [8]. The correlation between elevated cortisol levels and reduced rACC volume among depressed individuals is also consistent with results from animal studies regarding the role of mPFC in HPA axis negative feedback regulation. It is noteworthy that only the average of all seven cortisol samples was correlated with rACC volume, while the average of the morning samples was not. This suggests that volumetric decreases in the rACC are not specifically linked to peak cortisol activity; rather, rACC volume appears to be more closely related to sustained glucocorticoid exposure.

Our findings suggest that chronic stress subsequent to childhood maltreatment may serve to initiate glucocorticoid-related injury to the ACC. This damage may impair cortico-limbic circuits involved in emotion regulation; in addition, insult to the ACC may diminish its ability to exert negative feedback control over future HPA activity. Together, these two outcomes may result in poor regulation of stress, and could play a role in both the initiation of depression and increased vulnerability to recurrence. A recent longitudinal study also suggests that decreased volume in ACC in individuals with depression may result from stress [35]. Further research will be required to clarify the temporal relationships between early adverse events, increased HPA activity and structural integrity of the ACC.

We did not find any group differences in the hippocampus. This may result from heterogeneity of important clinical variables within our sample, including the number of episodes, duration of illness and severity of early life trauma. Prior studies that have identified hippocampal decreases associated with MDD have often reported that the extent of hippocampal damage is associated with the duration of illness [10], [12] particularly when it is untreated [11] (cf Campbell and MacQueen, 2006 for a review [15]). In contrast, the number of previous episodes in our sample ranged from none to four or more. Similarly, Vythilingam et al found volumetric decreases in individuals with both MDD and a history of severe child abuse, but not MDD alone [13]. In our sample, the severity of early adverse events varied from none to severe. This heterogeneity in the severity of early life stress may partially explain why we failed to observe group differences in hippocampal volume. Finally, it should be mentioned that voxel-based morphometry is not the ideal method for investigation of the hippocampus, as the anatomical complexities of this structure make it very difficult to accurately segment grey and white matter tissue classes using this technique.

### Limitations

Several limitations in the present study warrant mention. First, we did not find any significant differences using a whole-brain analysis after correcting for multiple comparisons, suggesting that where volumetric differences occurred in our depressed subjects, the effects sizes were only small to moderate. An additional limitation is the use of the VBM method, which is susceptible to normalization and segmentation errors. This issue is compounded by our relatively small sample size, as VBM is best suited for a sample size of 25 subjects or more per group. It should also be noted that this method is particularly susceptible to errors in the evaluation of hippocampal volume, and manual segmentation remains the preferred method for this region. Further, patients with MDD were asked to evaluate their history of traumatic childhood experiences while they were in the acute phase of depression, which may have influenced their memory for events. Our study was limited by its reliance on salivary cortisol as the only measure of HPA activity, as opposed to other forms of assessment of HPA function such as the dexamethasone suppression test or the corticotropin releasing hormone (CRH) test. Finally, the complete neurobiological mechanisms by which elevated cortisol precipitates structural damage in the ACC are likely to involve additional variables that were not evaluated in the present study.

### Conclusion

The present study replicates several previous reports suggesting the MDD is associated with decreased grey matter volume in the anterior cingulate. In addition, this study suggests that the extent of grey matter loss is related to both a history of early adverse events and circulating cortisol levels. These data further implicate the rostral cingulate as a key region in the regulation of HPA activity and the pathophysiology of MDD.
